# Exploring how to improve access to psychological therapies on acute mental health wards from the perspectives of patients, families and mental health staff: qualitative study

**DOI:** 10.1192/bjo.2022.513

**Published:** 2022-06-14

**Authors:** Katherine Berry, Jessica Raphael, Gillian Haddock, Sandra Bucci, Owen Price, Karina Lovell, Richard J. Drake, Jade Clayton, Georgia Penn, Dawn Edge

**Affiliations:** Division of Psychology and Mental Health, School of Health Sciences, Faculty of Biology, Medicine and Health, Manchester Academic Health Science Centre, The University of Manchester, UK; and Department of Research and Innovation, Greater Manchester Mental Health NHS Foundation Trust, UK; Division of Psychology and Mental Health, School of Health Sciences, Faculty of Biology, Medicine and Health, Manchester Academic Health Science Centre, The University of Manchester, UK; and Department of Research and Innovation, Greater Manchester Mental Health NHS Foundation Trust, UK; Division of Psychology and Mental Health, School of Health Sciences, Faculty of Biology, Medicine and Health, Manchester Academic Health Science Centre, The University of Manchester, UK; and Department of Research and Innovation, Greater Manchester Mental Health NHS Foundation Trust, UK; Division of Psychology and Mental Health, School of Health Sciences, Faculty of Biology, Medicine and Health, Manchester Academic Health Science Centre, The University of Manchester, UK; and Department of Research and Innovation, Greater Manchester Mental Health NHS Foundation Trust, UK; Division of Nursing, Midwifery and Social Work, School of Health Sciences, Faculty of Biology, Medicine and Health, Manchester Academic Health Science Centre, The University of Manchester, UK; Division of Nursing, Midwifery and Social Work, School of Health Sciences, Faculty of Biology, Medicine and Health, Manchester Academic Health Science Centre, The University of Manchester, UK; Division of Psychology and Mental Health, School of Health Sciences, Faculty of Biology, Medicine and Health, Manchester Academic Health Science Centre, The University of Manchester, UK; and Department of Research and Innovation, Greater Manchester Mental Health NHS Foundation Trust, UK; Department of Research and Innovation, Greater Manchester Mental Health NHS Foundation Trust, UK; Division of Psychology and Mental Health, School of Health Sciences, Faculty of Biology, Medicine and Health, Manchester Academic Health Science Centre, The University of Manchester, UK; Division of Psychology and Mental Health, School of Health Sciences, Faculty of Biology, Medicine and Health, Manchester Academic Health Science Centre, The University of Manchester, UK; and Department of Research and Innovation, Greater Manchester Mental Health NHS Foundation Trus, UK

**Keywords:** Psychosocial interventions, patients, qualitative research, in-patient treatment, cognitive–behavioural therapies

## Abstract

**Background:**

Psychological therapy is core component of mental healthcare. However, many people with severe mental illnesses do not receive therapy, particularly in acute mental health settings.

**Aims:**

This study identifies barriers to delivering and accessing psychological therapies in acute mental health settings, and is the first to recommend how services can increase access from the perspectives of different stakeholders (staff, patients and carers).

**Method:**

Sixty participants with experiences of acute mental health wards (26 staff, 22 patients and 12 carers) were interviewed about barriers to accessing therapy in in-patient settings and how therapies should be delivered to maximise access.

**Results:**

Four themes were identified: (a) ‘Models of care’, including the function of in-patient wards, beliefs about the causes of mental health problems and the importance of strong leadership to support psychosocial interventions; (b) ‘Integrated care’, including the importance of psychologists being ward-based, as well as having strong links with community teams; (c) ‘Acute levels of distress’, including factors that aggravate or ameliorate the impact of this on engagement in therapy; and (d) ‘Enhancing staff capability and motivation’, which is influenced by contextual issues.

**Conclusions:**

It is possible to improve access to therapy through strong leadership (that is supportive of talking treatments), flexible delivery of therapy (that considers short admissions) and a whole-systems approach that promotes ward staff understanding of the psychosocial causes of mental illness and staff well-being. It is essential to ensure continuity between in-patient and community therapy services, and for wards to have physical space to carry out therapy.

People with severe mental illness should have access to evidence-based psychological therapies, such as cognitive–behavioural therapy (CBT),^1,2^ but most people in the UK with severe mental illness do not receive them.^3^ Acute mental health settings pose particular barriers to the implementation of psychological therapies, and in the UK, there have been longstanding criticisms regarding the non-therapeutic nature of in-patient care.^4^ Challenges of delivering psychological interventions on acute wards in the UK and elsewhere include short in-patient stays, complexity of patient issues and presentation, and inadequate team working.^5–10^ It is important to build on previous research focused on challenges, and identify how therapies should be delivered in in-patient wards to improve access.^9^ Service change is challenging and often unwelcome. It is therefore important to involve stakeholders in consultation from beginning to end, which can increase a sense of ownership in the approach. This study considers how best to increase access to therapies on acute in-patient wards from the perspectives of staff, patients and carers.

## Method

### Design

This qualitative study was conducted from April 2018 to July 2019 across the UK. Data collection was by individual semi-structured interviews.

The authors assert that all procedures contributing to this work comply with the ethical standards of the relevant national and institutional committees on human experimentation and with the Helsinki Declaration of 1975, as revised in 2008. All procedures involving human patients were approved by the North West South NHS Research Ethics Committee (reference 18/NW/0009).

### Recruitment

Inclusion criteria were as follows: (a) staff with current experience of working in acute in-patient settings; (b) or people ≥18 years, with at least a week's experience of acute in-patient care over the previous 24 months; (c) and/or family members/carers who were carers for or living with people meeting criterion (b). Participants were recruited via National Health Service (NHS) services (community and wards), social media and flyers distributed via mental health charities. We developed a sampling frame involving demographic variables and purposefully sampled to help ensure key demographics were represented and until data saturation was reached.

### Data collection

Interview guides were developed in consultation with the study Patient and Public Involvement (PPI) group (which comprised ten former in-patients and/or carers) and explored views on care on acute mental health wards, access to psychological therapies and barriers/facilitators to access and delivery. All participants provided written informed consent before commencing interviews and were told that interviews focused on views about psychological therapy for in-patients. Four patients, who were initially consented to the research, declined when later approached to complete the interview. Interviews were audio-recorded and transcribed verbatim. Interviews were conducted by one of two female postgraduate researchers (including J.R.) either face to face or over the phone, and lasted 9–122 min (mean 40 min).

### Data analysis

Transcripts were coded within NVivo11 (QSR International Pty Ltd, Doncaster, Australia; see https://www.qsrinternational.com/nvivo-qualitative-data-analysis-software/home) and analysed with thematic analysis.^11^ Data from all stakeholder groups were coded together by J.R. The first ten transcripts were inductively coded and used to agree on a coding framework for subsequent analysis. The framework evolved throughout the project as new codes emerged. A random selection of transcripts were coded by D.E. and five PPI members, to check coding consistency. Codes were grouped into themes through discussions between J.R., D.E. and K.B. The organisation and labelling of themes were refined through discussion with the wider team. Practice recommendations resulting from the themes developed in the analysis were both drawn from the analysis and, as such, interwoven with the themes, or from discussions of the findings with the wider research team, including the PPI group.

## Results

A total of 26 staff, 22 patients and 12 carers were interviewed (see Table 1). Some themes were primarily reflected in staff data and others in patient/carer data. Similarities and differences in perspectives are highlighted below and in Table 2.

**Table 1:** Sample characteristics

	Total	*n*
Staff demographics		
Gender	Male	7
	Female	19
Age	18–25 years	2
	26–40 years	10
	41–60 years	14
Ethnicity	White British	20
	White other	2
	Mixed	1
	South Asian	2
	Chinese	1
Profession	Nursing assistant	4
	Ward manager	3
	Occupational therapist	3
	Assistant psychologist	1
	Clinical psychologist	6
	Psychiatrist	2
	Registered mental health nurse	7
Experience on ward	<1 year	2
	1–5 years	12
	5–10 years	2
	>10 years	10
Years since qualified	<10 years	11
	≥10 years	15
Region of UK recruited from	North-West	18
	Midlands	3
	South-East	3
	North-East	1
	Scotland	1
Patient demographics		
Gender	Male	6
	Female	16
Age	18–25 years	7
	26–40 years	7
	41–60 years	7
	≥61 years	1
Ethnicity	White British	16
	White other	2
	Mixed	1
	South Asian	2
	Black	1
Primary diagnosis	Schizophrenia	6
	Personality disorder	2
	Major depressive disorder	4
	Bipolar disorder	6
	Schizoaffective disorder	2
	Not known	2
Number of admissions	1	9
	2–5	7
	6–10	3
	>10	3
Previous access to therapy	In-patient	6
	Out-patient	10
	In-patient and out-patient	2
	None	4
Region of UK recruited from	North-West	16
	Midlands	2
	South-East	2
	North-East	2
Carer demographics		
Gender	Male	4
	Female	8
Age	41–60 years	6
	≥61 years	6
Ethnicity	White British	10
	White other	1
	Black	1
Primary diagnosis of person care for	Schizophrenia	3
	Personality disorder	1
	Major depressive disorder	2
	Bipolar disorder	3
	Other	3
Number of admissions for person care for	1	1
	2–5	5
	6–10	1
	>10	5
Previous access to therapy for person care for	In-patient	4
	Out-patient	6
Region of UK recruited from	None	2
	North-West	7
	South-East	2
	North-East	2
	Midlands	1

**Table 2:** Summary of themes and recommendations

Theme (perceptions and experiences related to themes)	Participant group	Implications	Recommendations
Models of care			
Acute wards function to stabilise mental states and contain immediate risk	Staff Patient	Patients are discharged as quickly as possible Lack of consideration given to other needs resulting in high rate of readmissions Psychological therapy not started or prioritised No dedicated therapy space	Senior staff need to promote psychosocial models/psychological approaches in terms of understanding all types of mental distress Psychologists need to be visible and present on the wards and involved in decisions about who would benefit from therapy Wards need dedicated therapy rooms
People with psychosis are not prioritised for therapy as problems, as they are believed to respond to medication	Staff	People with psychosis do not have access to the full range of evidence-based care throughout the care pathway	
Integrated care			
Psychologist not seen as core members of the multidisciplinary team	Staff	People who might benefit from therapy do not receive it Staff are not exposed to psychological theories of mental distress and do not benefit from the broader support that psychologist can provide to staff teams	Psychologists need to be ward based and frequently present on the wards
Lack of continuity between care on the ward and elsewhere (e.g. community, other services)	Staff Patient Carer	People do not start therapy because of concerns about work not continuing post discharge	Staff need to recognise the value of short-term therapies Staff need to develop good relationships with community teams so psychological formulations and therapies can be handed over
Acute levels of distress			
Patients’ acute state of distress affecting what can be offered and adaptations	Staff Patient Carer	People are not offered or do not uptake therapy who might benefit Patients do not know what therapies are available	Psychologists need to be aware of common motivational barriers, including those that might be unique to the in-patient setting and build alliances Therapy may be about containing emotions Psychologists should be based on the ward and talk to staff and patients about what can be provided Staff should talk to carers about what can be provided
Staff capability and motivation			
Staff can lack capability and motivation to support psychological therapies	Staff	Staff may not promote therapies to patients Staff may not use the knowledge and skills they do have to engage patients in therapeutic work	Ward staff should have regular training and supervision in psychological models of mental distress and how to deliver low-level psychological interventions (e.g. psychoeducation and coping skills enhancement) Psychologists should build supportive alliances with staff to help understand and overcome motivational barriers

### Theme 1: models of care

This theme was primarily within staff interviews and related to beliefs about the function of an in-patient ward, the causes of mental health problems and the consequent impact of these beliefs on the care provided. Participants reflected on how strong leaders who endorsed psychosocial models of mental health were pivotal in disengaging from a purely medical model.

When staff viewed the function of acute wards as the rapid stabilisation of mental state through medication and containment of risk, there was little room for psychological approaches, which were seen as secondary. For example, one nurse, reflecting on difficulties she experienced in advocating for psychological therapy, stated:

‘I think psychology takes a bit of a back burner because they think, we need to get the meds stable and get “them out”’ (Staff 06).

This view was echoed by a psychiatrist, who highlighted the importance of:

‘minimising the length of stay as much as possible, and thinking how soon someone can be discharged’ (Staff 09).

Pressure to rapidly stabilise and discharge patients quickly was partly driven by a need to free up beds for new admissions:

‘We have phone calls, throughout the day; bed management, asking us who's going, when are they going, why are they not going, what can we do to get them out?’ (Staff 24).

Although patients and carers did not see a length of stay as a reason not to offer therapy, they did perceive a drive toward quick discharge, which resulted in their needs not being met and lack of patient-centred care. As this patient reflected:

‘So many times, the discharge happened too early, and I was back again, so it's like a revolving door’ (Patient 11).

As wards were primarily functioning to stabilise acute mental states, they were in one nurse's words:

‘…very busy, chaotic places, just by the virtue of the needs of the patients, and often staff are caught up in terms of trying to support the most needy patients and, and reacting to situations’ (Staff 24).

There was a general consensus among all participant groups that the ward environment was busy and chaotic. For example, as one patient described:

‘The alarms go off quite a lot when other people are trying to hurt themselves, and it can disrupt other patients’ (Patient 10).

There was also often no dedicated rooms for psychological therapy on many wards, highlighting the lack of consideration of the potential importance of therapy within an acute admission. In reflecting where she would like therapy to be delivered, one patient stated quite simply that:

‘Having somewhere to go, like a quiet room, that would be quite good’ (Patient 06).

The focus on pharmacological treatment as opposed to therapy was particularly evident for patients with psychosis, who were understood as having a ‘mental illness’ that could be stabilised relatively quickly and treated effectively through medication. Conversely, patients with the label of ‘personality disorder’ were thought to have ‘behavioural problems’ that were within the person's own control and were unlikely to respond to medication. Staff who viewed the role of in-patient care in terms of medical treatments and containment, viewed patients with personality disorder as problematic, as they did not fit the traditional treatment model. As staff felt lost in knowing how to work with problems that did not respond well to medical treatment, the role of working with personality disorders often fell to psychologists.

In discussing referrals, one assistant psychologist stated:

‘People with emotionally unstable personality disorder are more likely to be referred to us because it's seen as something that only psychology can help’ (Staff 03).

Staff reflected that changing the perceived function of in-patient care and viewing problems with a psychological lens (regardless of diagnosis) required a shift in the centrality and visibility of psychologists from the admission process onward. For example, one psychologist remarked:

‘I would like to think that you got to the point where everybody, when they enter the ward, sees a psychologist in the same way as they see a psychiatrist, and that would be the starting point’ (Staff 10).

Participants also felt that the adoption of psychologically informed care was dependent on the commitment of senior nurses. For example, reflecting on what helped ward staff attend her formulation sessions, one psychologist said:

‘…having ward managers, who really value it, who basically tell the staff that it's part of their role, and they have to attend’ (Staff 02).

Other staff described how enthusiasm for psychological approaches among nursing staff could be overridden by psychiatrists who did not value this work:

‘The consultant will come in, they'll do the ward round. You'll have somebody in the ward round, normally a nurse, or a nursing assistant who's experienced, to feedback, but they often don't feel very listened to or if there's a difference of opinion there's no conversation about that’ (Staff 19).

These findings highlight the hierarchical nature of the ward environment and how changing the emphasis and focus of care, needs to come from system leaders as opposed to bottom up.

### Theme 2: integrated care

This theme reflected the importance of psychologists being ward-based for improving access to therapy. It also reflected the need for improved communication between in-patient teams and community services to ensure continuity of psychological care. This theme cut across interviews with all participant groups.

For patients and carers, quite simply, the absence of a psychologist attached to the ward meant that they did not have access to therapy. As one carer highlighted:

‘I understand that even now in this unit here, they haven't got a psychologist, so there's no therapy at all’ (Carer 05).

This suggests that psychologists in the community spent little time within the ward environment. Even when psychologists were ward-based, patients reflected that psychologists were not visible. For example, one patient remarked:

‘The psychologist only comes in every week, if requested, she's not proactive in her approach’ (Patient 22).

This lack of visibility may reflect findings in the staff data which suggested that psychologists were not seen as part of the core multidisciplinary team (MDT). In part, this may reflect a reluctance on the psychologist's part to integrate within the team because of a perception that the ward model of care contrasts with their own. However, this lack of integration could perpetuate the divide and differences in understanding. As this quote from a psychologist demonstrates, changes in practice were possible when psychologists appreciated the value of accessibility:

‘We've made a real effort to not lock ourselves away, which has been quite difficult at times, because it's really horrible on the wards sometimes, but we will always try and make time to sit in the nurses office, so that we're accessible’ (Staff 02).

Frequent contacts between psychologists and ward staff, and psychologists and patients, was regarded as important for increasing openness to psychological interventions and increasing awareness of the wide scope of the psychologist's role. Similarly, carers and patients felt that having psychologists more present on wards would help to increase patients’ confidence in engaging in talking therapies. For example, one carer reflected:

‘…building up a relationship is seeing somebody around, seeing that they're approachable, seeing that they're interacting, just being there and talking’ (Carer 05).

One patient described how seeing the psychologist running groups helped her to feel more confident in meeting the psychologist one-to-one:

‘I think having a group session helped and then he sees people one to one, so, having a group therapy is really good to break down any uncertainty’ (Patient 14).

These findings highlight the inherent mistrust patients may have in relation to mental health professionals, and how vital relationship building is in terms of breaking down this barrier.

On wards where psychologists were not fully integrated with the teams, patients accessed psychological therapy through referrals from ward staff. This represented a significant problem of some patients being overlooked, given the aforementioned biases in staff judgements of which patients were suitable for psychological therapy and negative attitudes toward in-patient therapy. It therefore underscores the need for psychologists to be integrated with the MDT and engaged in the referral process. As one psychologist reflected, staff who were not supportive of psychological approaches might discourage people from engaging in therapy:

‘You need staff who sell your services, and if people feel like your services may not be very useful, they're not gonna be able to sell them very well’ (Staff 01).

These barriers highlighted the need for psychologists to be integrated within the MDT and engaged in the referral process.

Psychologists were not only at risk of working as lone practitioners on the wards, but also not linking in with services outside of the ward. This problem was not specific to psychological work, but reflected a phenomenon highlighted by all participant groups about the ward in general. The ward was described as a microsystem, separate from other wards and community services, despite patients moving frequently between services. The problem was revealed in staff descriptions of patients being unable to complete work they had started in therapy once they had been transferred to another ward/community service:

‘*It's unfair on the service user to actually start that work then be discharged*’ (Staff 05).

‘*The frustrations can be that, the time that they're about to start the interventions is the time when actually they're starting to be discharged*’ (Staff 07).

These quotes indicate a lack of value placed by staff in the benefits of short-term therapy for in-patients, as well as a perceived lack of opportunity for collaborative working between in-patient and community services.

When staff recognised the value of sharing information between services, they emphasised the importance of handing over work from in-patient to community teams:

‘I think it's about engaging people in the community when you've got a pretty good idea that discharge is on the cards and making sure that you've got that, information up front and perhaps having a discussion with, psychologists in the community that can then pick up the work’ (Staff 10).

However, opportunities for continuing work in the community were often hindered by long waiting lists, suggesting it may not be possible for patients to continue seeing a therapist if therapy work was started on an in-patient ward. As highlighted by one psychiatrist:

‘We'd have to refer them to the CMHT [community mental health team]-based psychologist, but there was always a waiting list’ (Staff 9).

Like staff, patients and carers reflected on poor access to therapies in the community and follow up. For example, one patient described her disappointment that the groups were not continued following her discharge despite being informed that they would be:

‘It was a good therapy group, and there was meant to be about four sessions and around half way through I was discharged and was meant to come back but they never emailed me back about it’ (Patient 11).

This quote demonstrates how lack of follow-up can create disempowering experiences for patients, especially if the reasons are not communicated.

However, carers and patients did not see lack of community provision as reason not to offer therapy in in-patient settings, and some even spoke of the in-patient admission as being the ideal time for therapy. For example, one patient talked about how she was free from other life distractions in the in-patient environment so that she could focus more on therapy than if she had been an out-patient:

‘Psychology as an in-patient is more hands on, you get the sterile environment which kind of helps to a certain degree’ (Patient 22).

One carer felt that lack of access to therapy in the community reinforced a greater need for therapy in in-patient settings:

‘When they're in a ward setting and then they come out, they've lost some of that initial thing why it happened, and that's a missed opportunity, where they're still in the ward, it could be a quick intervention rather than having to wait and then you've lost some of that, explanation of why you've dealt with it in that way, ‘cos you've been waiting too long in the community’ (Carer 4).

### Theme 3: acute levels of distress

This theme reflects the impact of acute levels of distress on a patient's capacity to engage in psychological therapy, and factors which aggravate or ameliorate the impact of this distress. Concepts were reflected in interviews from all participant groups but, as highlighted below, there were key differences between staff and patient participants’ perceptions of patient capacity and willingness to engage in therapy.

As these quotes illustrate, all groups of participants reported patient difficulty concentrating and retaining information because of distress and/or medication side-effects as barriers to patients accessing therapy.

‘I think it is very difficult for service users, because they're often in a state of distress, and when you're very distressed it's very hard to take on board anything’ (Carer 09).

‘…depending on medication your concentration is just out the window’ (Patient 14).

‘When we're getting patients on our ward they can be very poorly and acutely unwell, and sometimes it can be difficult to have a really basic conversation with somebody’ (Staff 06).

Although most staff participants, including psychologists, acknowledged these difficulties, staff nevertheless felt that it was possible to offer patients some form of talking therapy if the goals of therapy can be adapted to the in-patient environment. As one assistant psychologist reflected:

‘People just need containing, they might not be able to think about moving forward, but they just might want someone, or need someone to sit with them and listen’ (Staff 03).

This need for containment was also echoed in the patients’ descriptions of therapy:

‘It's more getting stuff off me chest, like with things that have happened, like arguments’ (Patient 18).

Although the majority of patient participants wanted access to therapy on acute wards, they described fearing that engaging in therapy would result in them disclosing the full extent of their difficulties and staff extending their admission.

’I feel embarrassed to talk about, stuff that we've talked about, because I feel like if I do, then I wouldn't be let out of hospital’ (Patient 08).

Patients reporting regretting that they had spoken too openly about their experiences within therapy:

‘I don't think I did myself any favours, which is all gonna be documented and put on my file and make me sound like a bad parent’ (Patient 17).

These articulated fears highlight the power that mental health professionals have over patients’ lives and how this can affect trust and openness. Staff also highlighted lack of patient trust as a barrier to engagement with therapy, suggesting the need for psychologists to be sensitive to some of the issues patients may face and the consequent need to spend time relationship building. As this staff member reflected:

‘There probably are some patients that it would take a long time to actually build up any trust with them’ (Staff 9).

Patient and carer participants highlighted the need for clear information regarding what psychological therapy involves and its benefits to maximise uptake, suggesting not everyone is informed about the nature of therapy and how it might be delivered in in-patient settings. For example, one patient described wanting to know:

‘…what it involves and just talking about it, ‘cos I would like to know a bit more about it’ (Patient 15).

However, access to such information was not always available, reflecting issues of general disempowerment within in-patient care. For example, one patient described how she was not given any details of how to access the psychologist, which reflects the problem with the reliance of staff referrals highlighted above:

‘When I came in I wasn't given any phone numbers or information’ (Patient 12).

Carers also suggested that they could help inform patients of available psychological services and offer encouragement to patients to access therapy. For example, one carer reflects how carers can support patients in the decision-making process:

‘Maybe it could be done through an intermediary, through your parents, or, someone visiting you, just to size things up’ (Carer 10).

However, carers generally felt excluded from the ward environment and disempowered. As this carer reflects:

‘It's alright treating the service user, but you've got people at home who care for that person, who are going through mental anguish not knowing what is being done to help that person’ (Carer 01).

### Theme 4: staff capability and motivation

The final theme reflects staff capability and motivation to deliver, support or engage in psychological approaches, which was inherently influenced by contextual and organisational issues. This theme emerged from the staff interviews. In terms of delivering psychological approaches, staff identified ward staffs lack of skills and confidence as barriers. For example, as one nurse reflected:

‘I would feel nervous to do it, ‘cos we're not used to doing psychology with patients. I guess, we would feel a bit anxious about it’ (Staff 17).

Conversely, as these nursing staff participants describe, when ward staff receive training and supervision in psychological approaches, this helps to increase self-efficacy and increases the incentive for further training and support:

‘I think there should be basic psychological modules introduced in our compulsory trainings’ (Staff 12).

I think it would need to be part of their supervision and maybe even having group supervision with psychologists; making it like mandatory, as part of your supervision, to discuss what you're doing (Staff 6).

Lack of staff motivation, for example, as a consequence of burnout, was identified as a further barrier to the implementation of psychological approaches. In speaking of some of her colleagues, one nurse noted:

‘They're working in these services for years, you get a bit staid, they become a bit old and a bit, (sigh) yeah; maybe they've been in it too long’ (Staff 22).

Participants noted ward staff adopt a mentality characterised by the need to ‘keep going’ despite encountering traumatic events, which could reduce engagement with psychologist-delivered support mechanisms, such as supervision and reflective practice. As one therapist reflected:

‘I've witnessed situations where I've said I think you need to take a break [staff member], and she said no, no, I'll be alright, and she's just been punched in the face. So, there is a bit of a culture of just putting up with’ (Staff 13).

The psychologist's relationship-building skills (e.g. listening to concerns and expressions of empathy) were seen as key to enhancing staff motivation to engage. For example, in reflecting on people's motivation to work with the psychologist, this nurse reflects on how the psychologist supports staff:

‘She's also been very, supportive, you know, on a one-to-one basis. She will come up and say, I can see that you're being pulled in every direction, why don't you go and have a drink. Sometimes, that's all you need, someone just to say, let me make you a drink, I can see you're looking really stressed, and you're being pulled in all directions’ (Staff 21).

## Discussion

### Summary of findings

This study identified ways of improving access to psychological therapy on acute mental health wards from the perspective of staff, patients and carers. There were four key themes: (a) theme 1, models of care (including staff beliefs about the function of an in-patient ward, staff beliefs about the causes of mental health problems and the importance of strong leadership to support psychosocial interventions); (b) theme 2, the need for integrated care both within the ward and between the ward and outside services; (c) theme 3, acute levels of distress and the consequent need to rethink the purpose and function of therapy; and (d) theme 4, enhancing staff capability and motivation, which includes ward staff skills and motivation to support psychological work on the ward.

### Comparison with wider literature and recommendations

Our study found that acute wards were viewed as places to stabilise patients with pharmacological intervention and reduce immediate risk via containment. This contrasts with the notion that wards should be therapeutic,^12^ and that patients should have access to all evidence-based treatments throughout the care pathway.^1,2^ This is a concerning finding because there is evidence from patient participants in this study and in previous research to suggest that if patients’ needs are not met within an admission, then this can result in rapid readmission.^13,14^ Our findings highlighted the importance of strong leadership from senior staff who advocate psychosocial models of mental healthcare, in terms of changing models of care away from solely medical models. The importance of leadership in bringing about organisational change is consistent with theories of implementation science.^15^ More specifically in this context, our findings suggest that psychologists need to be frequently physically present on the ward, developing relationships with other staff and patients. Psychologists also need to assertively promote psychological models of care alongside other senior members of the MDT, such as ward managers and consultant psychiatrists. The importance of psychologists being assertive in advocating psychological approaches contrasts with previous research suggesting that psychologists may lack confidence in putting forward psychological formulations to their MDT colleagues.^16^ Our findings are therefore important in highlighting the need to focus on development of leadership skills within clinical psychology training and, equally, the need to incorporate increased emphasis on psychological approaches in the core training of other members of the MDT, including psychiatrists.

Consistent with previous research,^5^ there was a risk of people with psychosis in particular having more difficulty accessing psychological therapy. This is despite the fact that psychosocial factors such as childhood trauma and social adversity are risk factors for the development of psychosis,^17^ and CBT is an evidence-based approach for psychosis.^1^ CBT can also be specifically adapted for in-patient settings; adaptations include focusing on short-term goals such as crisis management, patient-led delivery in terms of duration and frequency of sessions, and involvement from the wider MDT.^5^ Our findings highlight the importance of the MDT being alert to this bias related to psychosis and the development of psychological formulations for all patients, to highlight psychosocial needs regardless of diagnosis.^18^

Consistent with previous research, staff felt that the short length of in-patient admissions and lack of opportunities for continued provision of therapy in the community were barriers to delivering therapy.^5^ Conversely, our findings went further, to suggest that these were not concerns shared by patients and carers. There is also evidence that brief interventions can be effective for people with severe mental illness, which suggests length of stay should not necessarily be a barrier.^19^ Previous research has shown that psychologists can adapt evidence-based approaches to the in-patient environment, which includes brief standalone interventions.^20^ However, further research is needed to evaluate the efficacy of these approaches in sufficiently powered, randomised controlled trials.^5^

All groups of participants reflected that the busyness of acute care settings could be a barrier for psychological interventions, echoing the previously highlighted importance of wards having dedicated therapy rooms.^21,22^ Factors that might affect patient willingness to engage in psychological therapies have also been identified by previous research, and include factors associated with symptoms, medication and negative beliefs about therapy.^23^ These factors are not unique to in-patient settings, although some may be more prominent barriers in these settings and require more consideration from staff, such as poor concentration owing to distress and/or medication and fears about disclosing information that could result in prolonged admission. Flexibility in delivery may help to surmount problems with concentration. We suggest that whole-team approaches, which recognise the role of the patient's past relationship experiences (including experiences of abuse) and the iatrogenic effects of the mental health system in leading patients to mistrust mental health professionals, may also help staff to be empathetic toward patients’ reluctance to engage, and ultimately encourage patients to open up about experiences.^18^ Despite patient concerns about therapy, studies of psychological therapy on acute mental health wards highlight good levels of engagement and therapeutic alliance comparable with out-patient therapy, suggesting that it is possible to overcome patient-related barriers to engagement.^24^

In line with previous research, even if psychologists themselves are present to deliver therapy, ward staff's lack of knowledge and/or confidence in psychological approaches, or staff burnout, affect their capacity to support psychological approaches.^21,25^ As highlighted by our participants, ongoing training and supervision in psychological models of mental distress would be an important intervention to enhance staff engagement in delivering or supporting psychological therapies. As highlighted by our participants, psychologists may also have a role in providing emotional support to staff, with the aim of improving staff well-being and capacity to support psychological therapies. However, psychologist-led initiatives to support staff well-being need to be delivered within the context of organisational structures and cultures that overtly value staff and the contributions that they make to patient care.

### Strengths and limitations

Our sample was large and drawn from across the UK, although we acknowledge that our finding may not generalise to other countries that have different models of in-patient care. Despite attempts to purposefully sample, there was a relatively high proportion of White British participants, meaning that the voices of those from Black and minority ethnic backgrounds might not be as well represented. It would be particularly important for future studies to capture the voices of Black participants, given that they may be particularly disadvantaged in terms of access to psychological therapy.^3^ Despite the low rates of access to therapy within the UK, it is also noteworthy that a relatively high proportion of the patient/carer sample had experiences of therapy. This may mean that our sample may not be representative, although as access to therapy was self-reported, we do not know if these participants had experience of evidenced-based psychological therapies such as CBT. All of the psychologists that we interviewed had experience of working in acute mental health settings. Future studies should also explore the barriers that community-based therapists might face in relation to in-patient working, given the need for more integrated care between in-patient and community settings. The qualitative methodology enabled participants to talk freely, and a range of positive and negative views about therapy and care were expressed. The interviewers were both researchers, unknown to participants and who emphasised their independence from the clinical teams. Nonetheless, it is possible that some participants may have withheld viewpoints from the interviewers over concerns about how the data might be used, given that it was funded by the NHS. A strength of the study was PPI involvement in the development of interview schedules and analysis. Their involvement in the analysis in particular helped to ensure that the codes generated were meaningful for patients and carers. The process of coding the data and discussing study findings within PPI meetings also enabled PPI groups to contribute to recommendations about how to improve access to therapy.

In conclusion, participants identified multifaceted barriers to implementing psychological therapies in acute in-patient wards, but on the whole, participants from all groups were committed to overcoming these barriers and were able to highlight ways of doing so. These ideas provide clear recommendations for how those involved in acute care pathways can work together to enhance patient experience of acute care (see Table 2). In terms of future research, we need to empirically assess the added value of psychological therapy in in-patient wards in terms of patient outcomes, staff well-being and reductions in service costs.

## Data availability

The data that support the findings of this study are available on request from the corresponding author, K.B. The data are not publicly available due to their containing information that could compromise the privacy of research participants.

## References

[1] National Institute for Health and Care Excellence. Schizophrenia: Core Interventions in the Treatment and Management of Schizophrenia in Primary and Secondary Care (Update). National Institute for Health and Care Excellence, 2014.

[2] Penfold N, Nugent A, Clarke H, Colwill A. Standards for Acute Inpatient Services for Working Age Adults (7th edn). Royal College of Psychiatrists, 2019 (https://www.rcpsych.ac.uk/docs/default-source/improving-care/ccqi/quality-networks/working-age-wards-aims-wa/standards-for-acute-inpatient-services-for-working-age-adults—7th-edition.pdf?sfvrsn=acd9289c_2).

[3] Colling C, Evans L, Broadbent M, Chandran D, Craig TJ, Kolliakou A, Identification of the delivery of cognitive behavioural therapy for psychosis (CBTp) using a cross-sectional sample from electronic health records and open-text information in a large UK-based mental health case register. BMJ Open 2017; 7: e015297. 10.1136/bmjopen-2016-015297 PMC5734297 28716789

[4] Staniszewska S, Mockford C, Chadburn G, Fenton SJ, Bhui K, Larkin M, Experiences of in-patient mental health services: systematic review. Br J Psychiatry 2019; 214(6): 329–38. 30894243 10.1192/bjp.2019.22

[5] Wood L, Williams C, Billings J, Johnson S. Psychologists’ perspectives on the implementation of psychological therapy for psychosis in the acute psychiatric inpatient setting. Qual Health Res 2019; 29(14): 2048–56. 31014190 10.1177/1049732319843499

[6] Awenat Y, Peters S, Shaw-Nunez E, Gooding P, Pratt D, Haddock, G. Staff experiences and perceptions of working with in-patients who are suicidal: qualitative analysis. Br J Psychiatry 2017; 211(2): 103–8. 28642259 10.1192/bjp.bp.116.191817 PMC5537568

[7] Wood L, Williams C, Billings J, Johnson S. The therapeutic needs of psychiatric in-patients with psychosis: a qualitative exploration of patient and staff perspectives. BJPsych Open 2019; 5(3): e45. 31530314 10.1192/bjo.2019.33 PMC6537445

[8] Wood L, Williams C, Billings J, Johnson S. The role of psychology in a multidisciplinary psychiatric inpatient setting: perspectives from the multidisciplinary team. Psychol Psychother 2019; 92(4): 554–64. 30311351 10.1111/papt.12199

[9] Evlat G, Wood L, Glover N. A systematic review of the implementation of psychological therapies in acute mental health inpatient settings. Clin Psychol Psychother 2021; 28(6): 1574–86. 33870590 10.1002/cpp.2600

[10] Porter R, Averil I, Beaglehole B, Crowe M, Jordan J. Inpatient treatment for mood disorders – a lost opportunity? Aust N Z J Psychiatry 2016; 50(1): 7–8. 26706857 10.1177/0004867415616500

[11] Braun V, Clarke V. Successful Qualitative Research: A Practical Guide for Beginners. Sage Publications, 2013.

[12] Crisp N, Smith G, Nicholson K (eds). *Old Problems, New Solutions:* *Improving Acute Psychiatric Care for Adults in England*. The Commission on Acute Adult Psychiatric Care, Royal College of Psychiatrists, 2016 (https://www.rcpsych.ac.uk/docs/default-source/improving-care/better-mh-policy/policy/policy-old-problems-new-solutions-caapc-report-england.pdf?sfvrsn=7563102e_2).

[13] Phillips C, Tai S, Berry K. Experiences of acute mental health inpatient care in the UK: from admission readmission. Psychosis 2022; 14: 22–33.

[14] Osborn DP, Favarato G, Lamb D, Harper T, Johnson S, Lloyd-Evans B, Readmission after discharge from acute mental healthcare among 231 988 people in England: cohort study exploring predictors of readmission including availability of acute day units in local areas. BJPsych Open 2021; 7(4): e136. 34275509 10.1192/bjo.2021.961 PMC8329766

[15] May C, Finch T. Implementing, embedding, and integrating practices: an outline of normalization process theory. Sociology 2009; 43: 535–53.

[16] Christofides S, Johnstone L, Musa M. Chipping in: clinical psychologists descriptions of their use of formulation in multidisciplinary team working. Psychol Psychother 2012; 85(4): 424–35. 23080531 10.1111/j.2044-8341.2011.02041.x

[17] Varese F, Smeets F, Drukker M, Lieverse R, Lataster T, Viechtbauer W, Childhood adversities increase the risk of psychosis: a meta-analysis of patient-control, prospective- and cross-sectional cohort studies. Schizophr Bull 2012; 38(4): 661–71. 22461484 10.1093/schbul/sbs050 PMC3406538

[18] Berry K, Haddock G, Kellett S, Roberts C, Drake R, Barrowclough C. Feasibility of ward-based psychological intervention to improve staff and patient relationships in psychiatric rehabilitation settings. Br J Clin Psychol 2016; 55: 236–52. 25884235 10.1111/bjc.12082

[19] Hazell C, Hayward M, Cavanagh K, Strauss C. A systematic review and meta-analysis of low intensity CBT for psychosis. Clin Psychol Rev 2016; 45: 183–92. 27048980 10.1016/j.cpr.2016.03.004

[20] Folke F, Hursti T, Tungström S, Söderberg P, Kanter JW, Kuutmann K, Behavioral activation in acute inpatient psychiatry: a multiple baseline evaluation. J Behav Ther Exp Psychiatry 2015; 46: 170–81. 25460264 10.1016/j.jbtep.2014.10.006

[21] Raphael J, Price O, Hartley S, Haddock G, Bucci S, Berry K. Overcoming barriers to implementing ward-based psychosocial interventions in acute inpatient mental health settings: a meta-synthesis. Inter J Nurs Stud 2021; 115: 103870. 10.1016/j.ijnurstu.2021.103870 33486388

[22] Csipke E, Papoulias C, Vitoratou S, Williams P, Rose D, Wykes T. Design in mind: eliciting service user and frontline staff perspectives on psychiatric ward design through participatory methods. J Ment Health 2016; 25(2): 114–21. 26886239 10.3109/09638237.2016.1139061 PMC4819846

[23] Ince P, Haddock G, Tai S. A systematic review of the implementation of recommended psychological interventions for schizophrenia: rates, barriers, and improvement strategies. Psychol Psychother 2016; 89(3): 324–50. 26537838 10.1111/papt.12084

[24] Haddock G, Pratt D, Gooding P, Peters S, Emsley R, Evans E, Feasibility and acceptability of suicide prevention therapy on acute psychiatric wards: randomised controlled trial. BJPsych Open 2019; 5(1): e14. 30762509 10.1192/bjo.2018.85 PMC6381415

[25] Laker C, Cella M, Callard F, Wykes T. Why is change a challenge in acute mental health wards? A cross-sectional investigation of the relationships between burnout, occupational status and nurses’ perceptions of barriers to change. Inter J Ment Health Nurs 2019; 28(1): 190–8. 10.1111/inm.12517 PMC7328713 29993168

